# Characterization of the *Gh4CL* gene family reveals a role of *Gh4CL7* in drought tolerance

**DOI:** 10.1186/s12870-020-2329-2

**Published:** 2020-03-23

**Authors:** Shi-Chao Sun, Xian-Peng Xiong, Xiao-Li Zhang, Hong-Jie Feng, Qian-Hao Zhu, Jie Sun, Yan-Jun Li

**Affiliations:** 1 Key Laboratory of Oasis Eco-agriculture, College of Agriculture, Shihezi University, Shihezi, 832000 Xinjiang China; 2 Key Laboratory of Cotton Biology, Institute of Cotton Research, Chinese Academy of Agricultural Sciences, Anyang, 455000 Henan China; 3 CSIRO Agriculture and Food, GPO Box 1700, Canberra, 2601 Australia

**Keywords:** *Gossypium hirsutum*, 4CL, Transgenic *Arabidopsis*, Drought stress, VIGS

## Abstract

**Background:**

The function of 4-coumarate-CoA ligases (4CL) under abiotic stresses has been studied in plants, however, limited is known about the *4CL* genes in cotton (*G. hirsutum* L.) and their roles in response to drought stress.

**Results:**

We performed genome-wide identification of the *4CL* genes in *G. hirsutum* and investigated the expression profiles of the identified genes in various cotton tissues and in response to stress conditions with an aim to identify *4CL* gene(s) associated with drought tolerance. We identified 34 putative *4CL* genes in *G. hirsutum* that were clustered into three classes. Genes of the same class usually share a similar gene structure and motif composition. Many *cis*-elements related to stress and phytohormone responses were found in the promoters of the *Gh4CL* genes. Of the 34 *Gh4CL* genes, 26 were induced by at least one abiotic stress and 10 (including *Gh4CL7*) were up-regulated under the polyethylene glycol (PEG) simulated drought stress conditions. Virus-induced gene silencing (VIGS) in cotton and overexpression (OE) in *Arabidopsis thaliana* were applied to investigate the biological function of *Gh4CL7* in drought tolerance. The *Gh4CL7*-silencing cotton plants showed more sensitive to drought stress, probably due to decreased relative water content (RWC), chlorophyll content and antioxidative enzyme activity, increased stomatal aperture, and the contents of malondialdehyde (MDA) and hydrogen peroxide (H_2_O_2_). *Arabidopsis* lines overexpressing *Gh4CL7*, however, were more tolerant to drought treatment, which was associated with improved antioxidative enzyme activity, reduced accumulation of MDA and H_2_O_2_ and up-regulated stress-related genes under the drought stress conditions. In addition, compared to their respective controls, the *Gh4CL7*-silencing cotton plants and the *Gh4CL7-*overexpressing *Arabidopsis* lines had a ~ 20% reduction and a ~ 10% increase in lignin content, respectively. The expression levels of genes related to lignin biosynthesis, including *PAL*, *CCoAOMT*, *COMT*, *CCR* and *CAD*, were lower in *Gh4CL7*-silencing plants than in controls. Taken together, these results demonstrated that *Gh4CL7* could positively respond to drought stress and therefore might be a candidate gene for improvement of drought tolerance in cotton.

**Conclusion:**

We characterized the *4CL* gene family in upland cotton and revealed a role of *Gh4CL7* in lignin biosynthesis and drought tolerance.

## Background

Cotton is an important cash crop in many developing countries and frequently grown in dry lands or on supplementary irrigation [1], because agricultural water consumption can no longer be expanded thanks to water competition among domestic, industrial and agricultural users [2]. The quantity and quality of fiber produced by cotton plants are directly related to water available to them during their developmental stages. When suffered from water deficits, especially during the period of flowering and fructification, cotton would show significant yield loss, sometimes up to 50% reduction compared to those that have been irrigated [3, 4].

In the long-term evolutionary history, plants have formed a complex gene-metabolic network to accommodate a variety of environmental changes. As an important metabolite, lignin plays vital roles in defense against biotic and abiotic stresses [5–7]. Lignin is synthesized through the phenylpropane pathway. 4-coumarate-CoA ligases (4CL, EC 6.2.1.12) is the main branch point enzyme of the phenylpropanoid pathway, which catalyzes cinnamic acid to generate corresponding CoA thioesters [8]. Products of 4CL subsequently serve as substrates of various oxygenases, reductases and transferases for biosynthesis of lignin, flavonoids, anthocyanins, aurones, stilbenes, coumarins, suberin, cutin, sporopollenin, and others [9]. The *4CL* gene family has been characterized in many plants, such as *Arabidopsis* [10], rice [11] and aspen [12]. Genes of the *4CL* family in dicots can be classified into two distinct groups, type I and type II [8]. Type I genes are mainly involved in lignin biosynthesis whereas type II genes are involved in biosynthesis of phenylpropanoids other than lignin. Some additional genes containing the same conserved motifs of *4CLs* and showing high similarity with the 4CL proteins are classified as *4CL-like* genes [13].

Studies have shown that *4CL* genes play momentous roles in plants, such as regulation of growth and development, protection against biotic and abiotic stresses [11, 14, 15]. In *Arabidopsis*, *At4CL1*, *At4CL2*, and *At4CL4* were found to be involved in lignin formation, the *4 cl1 4 cl2* double and *4 cl1 4 cl2 4 cl3* triple mutant plants exhibited a dwarf and bushy phenotype [10]. In rice, *Os4CL2* was specifically expressed in anthers and induced by UV irradiation [16]. *Plagiochasma appendiculatum thallus* plants showed downregulation of *Pa4CL1* when treated with abscisic acid (ABA), and showed upregulation of *Pa4CL1* when treated with salicylic acid and MeJA [17]. In both poplars and *Arabidopsis*, the expression levels of *4CL* genes were induced by salt stress and wounding [7]. The *4CL-like* genes may also play a role in response to abiotic stresses [18, 19]. Overexpression of *Fm4CL-like1* in tobacco increased drought tolerance due to increasing lignin accumulation and the activities of antioxidant enzymes, and upregulating the expression levels of stress-related genes [19]. Nevertheless, our knowledge of the *4CL* gene family in cotton is very limited.

To gain insights into the cotton *4CL* gene family and its role in abiotic stress tolerance, in this study, we did genome-wide identification of *4CL* genes in *G. hirsutum* and analysed their expression changes in response to various abiotic stresses based on publically available RNA-seq datasets. We identified 34 putative *Gh4CL* genes in *G. hirsutum* and 26 of them were found to be induced by at least one stress, including *Gh4CL7* that was up-regulated under polyethylene glycol (PEG) osmotic stress. We further investigated the function of *Gh4CL7* in drought tolerance by silencing its expression in cotton using virus-induced gene silencing (VIGS) and generating transgenic *Arabidopsis* plants overexpressing *Gh4CL7*. Our results indicated that *Gh4CL7* functions positively in response to drought stress and is a potential candidate gene for improving drought resistance of cotton by genetic engineering.

## Results

### Genome-wide identification and bioinformatics analysis of *Gh4CL* genes

**Fig. 1:**
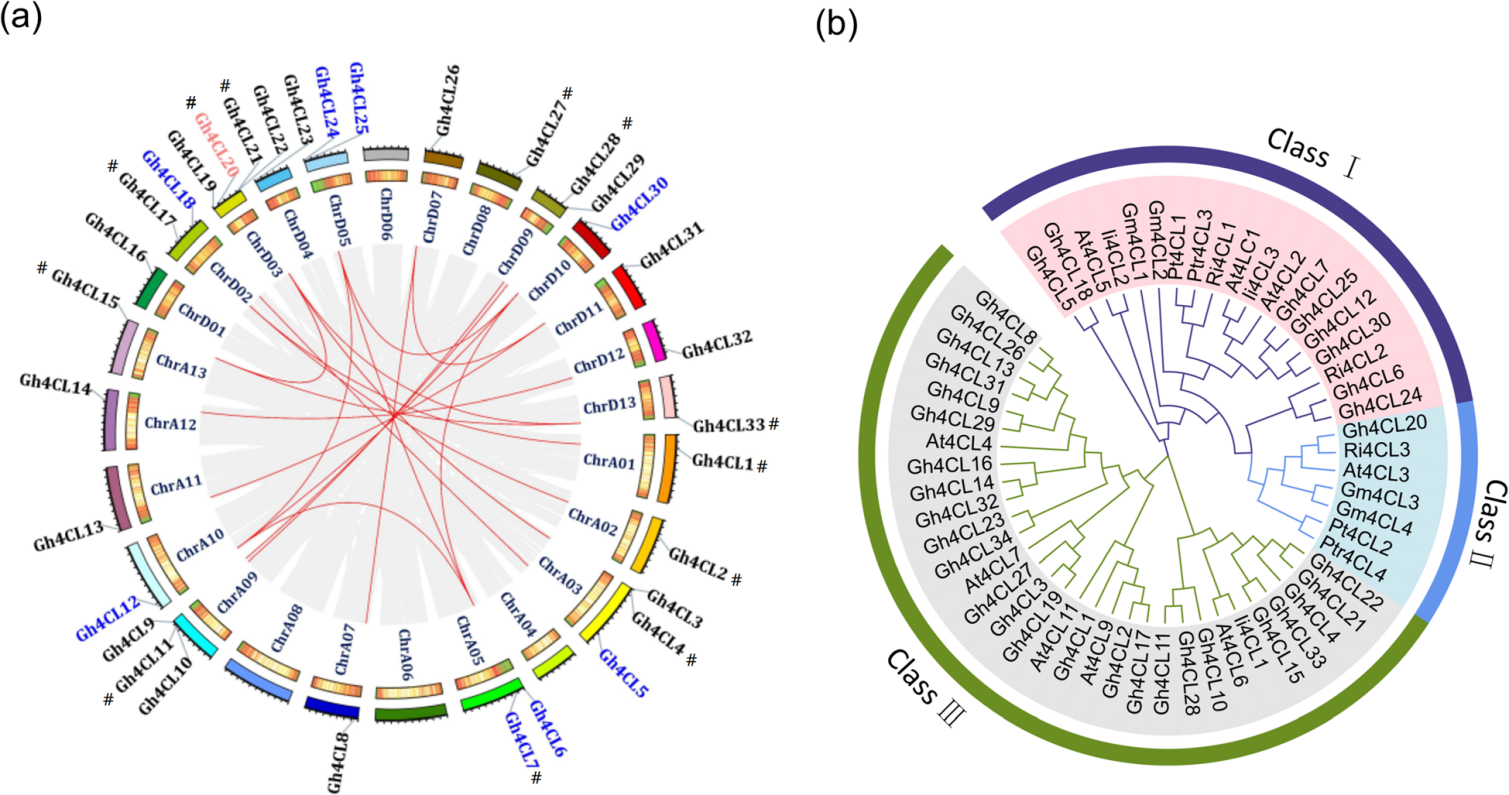
Phylogenetic, chromosomal distribution and interchromosomal relationships of the *Gh4CL* genes. **a** Chromosomal distribution of *Gh4CL* genes. The gray lines indicate all synteny blocks in the genome of *G. hirsutum* and the red lines indicate interchromosomal relationships of *Gh4CLs*. The first circle represents individual chromosomes length. The second circle represents the gene density of each chromosome by color-coded short bars. The chromosome number were shown inside the circle. **b** The neighbor-joining phylogenetic tree was generated using 4CL protein sequences from *G. hirsutum* and six other plants

Using the approach described in Materials and Methods, we identified 34 *Gh4CL* genes in *G. hirsutum*. They are randomly distributed on 22 chromosomes and an unanchored scaffold that was not assigned to a particular chromosome (Fig. 1a, Table 1). We named them *Gh4CL1* to *Gh4CL34* based on their chromosomal location. Two pairs of *Gh4CL* genes, *Gh4CL10/11* and *Gh4CL21/22*, are in tandem configuration on chromosomes A09 and D03, respectively. Segmental duplication could be involved in generation of 12 *Gh4CLs* based on MCScanX analysis. Phylogenetic analysis using the 34 *Gh4CL* genes and *4CL* genes from *A. thaliana*, *G. max*, *P. tremuloides*, *P. trichocarpa*, *R. idaeus*, and *I. tinctoria* showed that they were clustered into three groups (Fig. 1b). The Ka/Ks ratio of each homologous/paralogous *Gh4CL* pair is < 1 (Additional file 2: Table S1), suggesting that these *Gh4CL* genes have experienced purifying selective pressure during their evolution history to eliminate deleterious mutations.

**Table 1:** Characteristics of the 34 *Gh4CL* genes in *G. hirsutum*

Genes	Gene ID number	Genbank accession	Length/aa	MW/KDa	pI	subcellular localization
*Gh4CL1*	Gh_A01G0870	MN897786	552	60.36	8.66	mitochondrial inner membrane
*Gh4CL2*	Gh_A02G0862	MN897787	549	59.56	8.70	microbody (peroxisome)
*Gh4CL3*	Gh_A03G1962	MN897788	458	50.34	6.35	chloroplast thylakoid membrane
*Gh4CL4*	Gh_A03G0249	MN897789	517	57.08	8.32	plasma membrane
*Gh4CL5*	Gh_A03G1091	MN897790	555	61.09	5.73	plasma membrane
*Gh4CL6*	Gh_A05G3997	MN897791	543	59.56	5.67	plasma membrane
*Gh4CL7*	Gh_A05G1188	MN897792	543	59.51	5.84	endoplasmic reticulum (membrane)
*Gh4CL8*	Gh_A07G0468	MN897793	555	61.02	8.75	microbody (peroxisome)
*Gh4CL9*	Gh_A09G2180	MN897794	557	60.84	6.80	microbody (peroxisome)
*Gh4CL10*	Gh_A09G1370	MN897795	129	14.11	9.77	endoplasmic reticulum (membrane)
*Gh4CL11*	Gh_A09G1371	MN897796	459	50.53	8.54	plasma membrane
*Gh4CL12*	Gh_A10G0456	MN897797	543	59.61	5.30	endoplasmic reticulum (membrane)
*Gh4CL13*	Gh_A11G0333	MN897798	550	60.16	7.65	microbody (peroxisome)
*Gh4CL14*	Gh_A12G1362	MN897799	521	56.42	8.30	microbody (peroxisome)
*Gh4CL15*	Gh_A13G2028	MN897800	545	60.08	8.97	plasma membrane
*Gh4CL16*	Gh_D01G1584	MN897801	524	54.36	7.16	plasma membrane
*Gh4CL17*	Gh_D02G0989	MN897802	576	62.98	8.66	microbody (peroxisome)
*Gh4CL18*	Gh_D02G1514	MN897803	555	61.10	5.80	plasma membrane
*Gh4CL19*	Gh_D03G1840	MN897804	572	63.08	6.63	chloroplast thylakoid membrane
*Gh4CL20*	Gh_D03G0479	MN897805	573	62.04	5.54	plasma membrane
*Gh4CL21*	Gh_D03G1317	MN897806	452	49.64	8.40	plasma membrane
*Gh4CL22*	Gh_D03G1318	MN897807	517	56.97	8.79	plasma membrane
*Gh4CL23*	Gh_D04G0054	MN897808	568	61.94	6.17	microbody (peroxisome)
*Gh4CL24*	Gh_D05G3934	MN897809	540	59.26	5.90	endoplasmic reticulum (membrane)
*Gh4CL25*	Gh_D05G1366	MN897810	543	59.42	5.71	endoplasmic reticulum (membrane)
*Gh4CL26*	Gh_D07G0533	MN897811	555	61.10	8.78	microbody (peroxisome)
*Gh4CL27*	Gh_D08G1670	MN897812	559	61.33	6.60	microbody (peroxisome)
*Gh4CL28*	Gh_D09G1372	MN897813	546	60.17	8.66	plasma membrane
*Gh4CL29*	Gh_D09G2385	MN897814	557	60.94	6.50	microbody (peroxisome)
*Gh4CL30*	Gh_D10G0473	MN897815	543	59.52	5.42	endoplasmic reticulum (membrane)
*Gh4CL31*	Gh_D11G0389	MN897816	550	60.01	7.19	microbody (peroxisome)
*Gh4CL32*	Gh_D12G1488	MN897817	547	59.54	8.74	microbody (peroxisome)
*Gh4CL33*	Gh_D13G2431	MN897818	545	60.04	8.97	plasma membrane
*Gh4CL34*	Gh_Sca008083G01	MN897819	568	61.77	6.09	microbody (peroxisome)

The protein length of Gh4CLs is between 129 and 576 amino acids (aa) with ORF from 390 to 1731 bp, molecular weight from 14.11 to 63.08 KD, and pI from 5.3 to 9.77. Most Gh4CLs seem to be associated with various biomembranes based on subcellular localization prediction (Table 1). Analyses of gene structures and motifs showed that each *Gh4CL* has multiple exons, introns and motifs (Additional file 1: Figure S1; Additional file 2: Table S2). All the Gh4CL proteins have two structural domains, a putative AMP-binding domain “SSGTTGLPKG” (Box I) and a conserved domain “GEICIRG” (Box II) (Additional file 1: Figure S2).

*Cis*-elements in combination with transcription factors regulate the transcription level of a gene. To identify potential *cis*-elements involved in regulation of transcription of *Gh4CL* genes, we scanned *cis*-elements in the promoter region (2 kb upstream of ATG) of each *Gh4CL* gene using the online tool PlantCARE [20]. Many *Gh4CL* genes harbored plant hormone-responsive and/or stress-responsive elements, including ABA responsive elements (ABREs), auxin responsive elements (AuxRR-core, TGA-elements and TGA-box), MeJA-responsive elements (CGTCA-motif, TGACG-motif), gibberellin-responsive elements (TATC-box, GARE-motif and P-box), salicylic acid responsive elements (TCA-elements), low-temperature responsive elements (LTR), defense and stress responsiveness elements (TC-rich repeats) and drought-responsive elements (MBS) (Additional file 1: Figure S3).

### Tissue specific expression patterns of *Gh4CL* genes

**Fig. 2:**
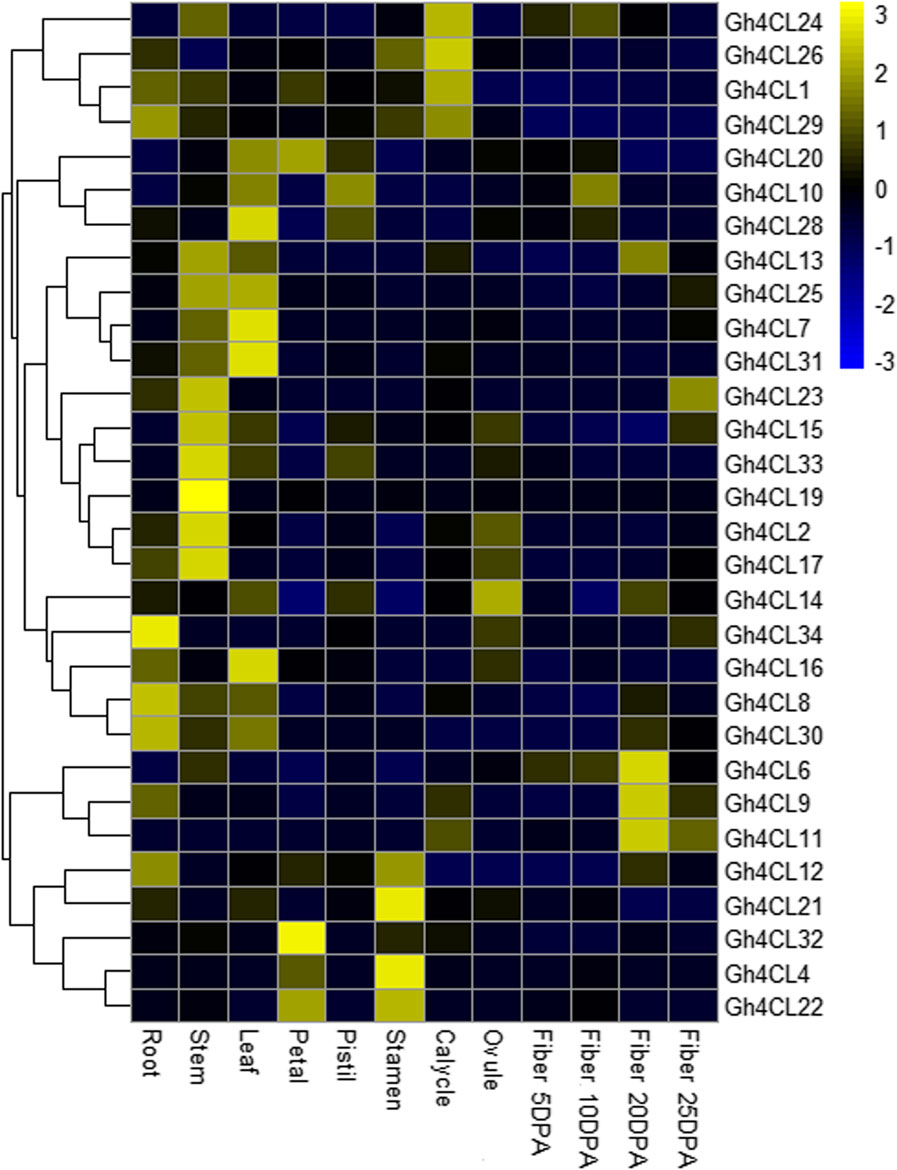
Expression pattern of the *Gh4CL* genes in different tissues of *G. hirsutum* based on the RNA-seq data. The heatmap was generated by using the pheatmap package

The expression patterns in various tissues provide clue for the possible biological functions of genes of interest. We thus analysed the transcript abundance of the *Gh4CL* genes in different tissues (root, stem, leaves, flower, ovule and fibers at 5, 10, 15 and 20 days-post-anthesis (DPA)) under normal growth conditions using the publically available RNA-seq data (BioProject Accession: PRJNA248163) [21]. We found that 10 *Gh4CL* genes were expressed in all the tested tissues [base on fragments per kilobase of transcript per million mapped reads (FPKM) ≥ 1], and 4 genes (*Gh4CL3*, *Gh4CL5*, *Gh4CL18* and *Gh4CL27*) showed weak or no expression in all tissues analysed (Fig. 2). In addition, 8 *Gh4CL* genes (*Gh4CL2*, *Gh4CL4*, *Gh4CL8*, *Gh4CL12*, *Gh4CL17*, *Gh4CL24*, *Gh4CL29* and *Gh4CL30*) were highly expressed (FPKM ≥20) in stem, with the highest expression level observed for *Gh4CL17* (FPKM ≥84) and 6 genes (*Gh4CL7–8*, *Gh4CL12*, *Gh4CL20* and *Gh4CL30–31*) were strongly expressed in leaves, with the highest expression level observed for *Gh4CL20* (FPKM ≥202).

### Expression analysis of *Gh4CL* genes under different abiotic stress conditions

**Fig. 3:**
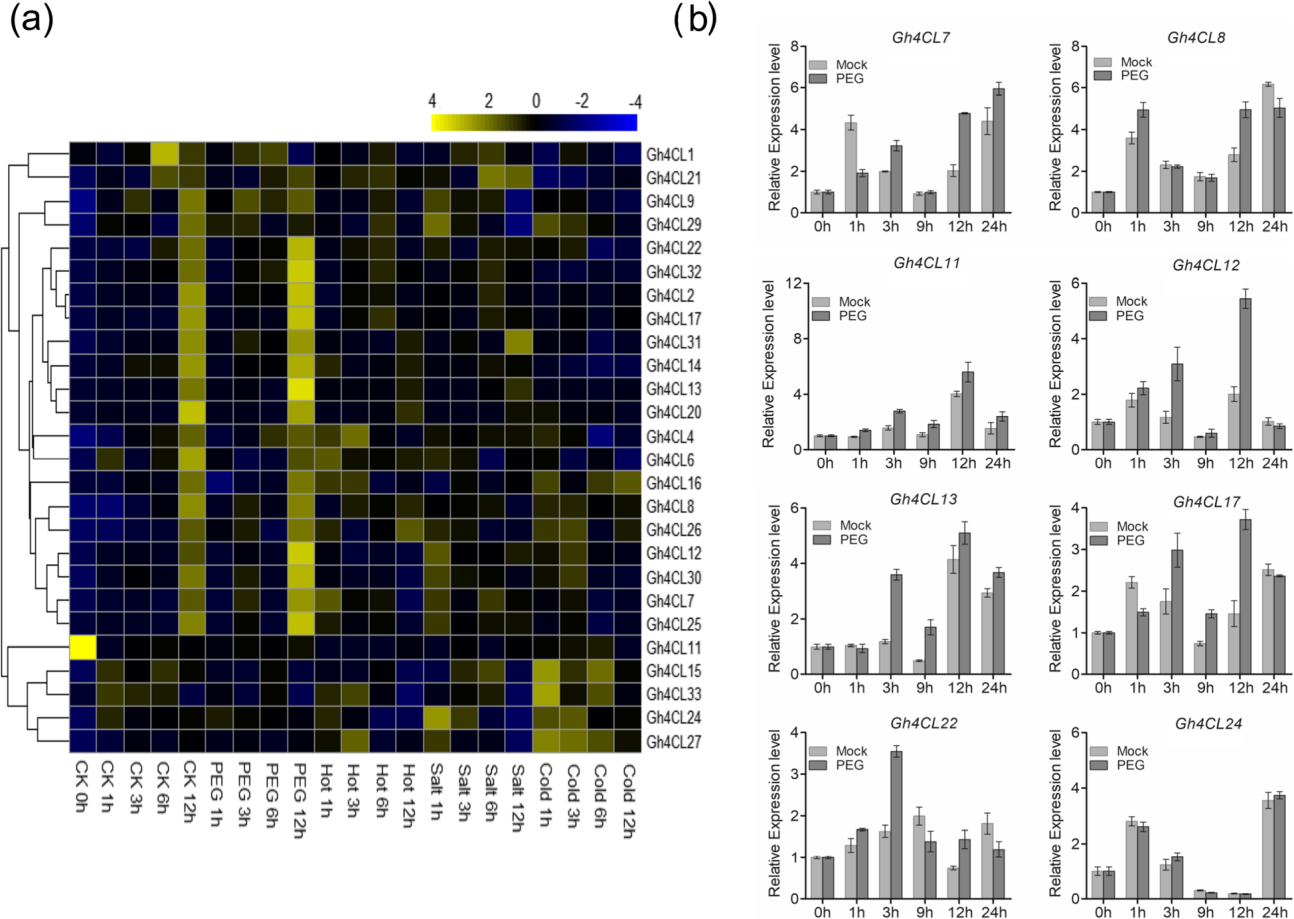
Expression profile of the *Gh4CL* genes in response to different abiotic stresses. **a** Heatmap showing the relative expression levels of each *Gh4CL* gene in each treatment showing at the bottom of the image. **b** The relative expression level of the selected *Gh4CL* genes under 20% PEG stress based on qRT-PCR. The *GhUBQ7* gene was used as internal control. Error bars represents the standard deviation calculated from three biological replicates

Since *4CL* genes are capable of responding to biotic and abiotic stresses in various plant species, we further investigated the transcript abundance of the *Gh4CL* genes under cold, heat, PEG and salt stresses using the transcriptomic data of *G. hirsutum* (BioProject Accession: PRJNA248163) [21]. We found that 26 *Gh4CL* genes were induced significantly by one or more stresses, and the remaining 8 *Gh4CL* genes (*Gh4CL3*, *Gh4CL5*, *Gh4CL10*, *Gh4CL18–19*, *Gh4CL23*, *Gh4CL28* and *Gh4CL34*) were not induced by either of the four stresses (Fig. 3a). Comparing the four stress conditions, more *Gh4CL* genes showed altered expression in response to salinity, cold and heat stresses than to PEG stress. Notably, ten *Gh4CL* genes (*Gh4CL2*, *Gh4CL7–9*, *Gh4CL11–13*, *Gh4CL17*, *Gh4CL22*, *Gh4CL25* and *Gh4CL31*) showed increased expression (treatment FPKM/control FPKM ≥1.5) in response to PEG stress over the 3 h to 12 h time period. To verify these results, we investigated the expression patterns of the selected *Gh4CL* genes under the simulated drought treatment using quantitative real-time polymerase chain reaction (qRT-PCR). As shown in Fig. 3b, the expression levels of *Gh4CL7*–*8*, *Gh4CL12*–*13*, *Gh4CL17*, *Gh4CL22* and *Gh4CL24* were up-regulated in cotton leaves over the time period of 3 h to 24 h after PEG stress, consistent with the RNA-seq based results.

### *Gh4CL7* plays an important role in lignin biosynthesis

Based on the above analysis results of promoter *cis*-elements and expression patterns under drought stress, three *Gh4CL* genes, including *Gh4CL7, Gh4CL8* and *Gh4CL13*, were considered as candidate genes with a potential role in the regulation of drought stress response in cotton. In this study, we selected *Gh4CL7* for further functional analysis by silencing its expression in cotton and overexpression in *Arabidopsis thaliana*.

**Fig. 4:**
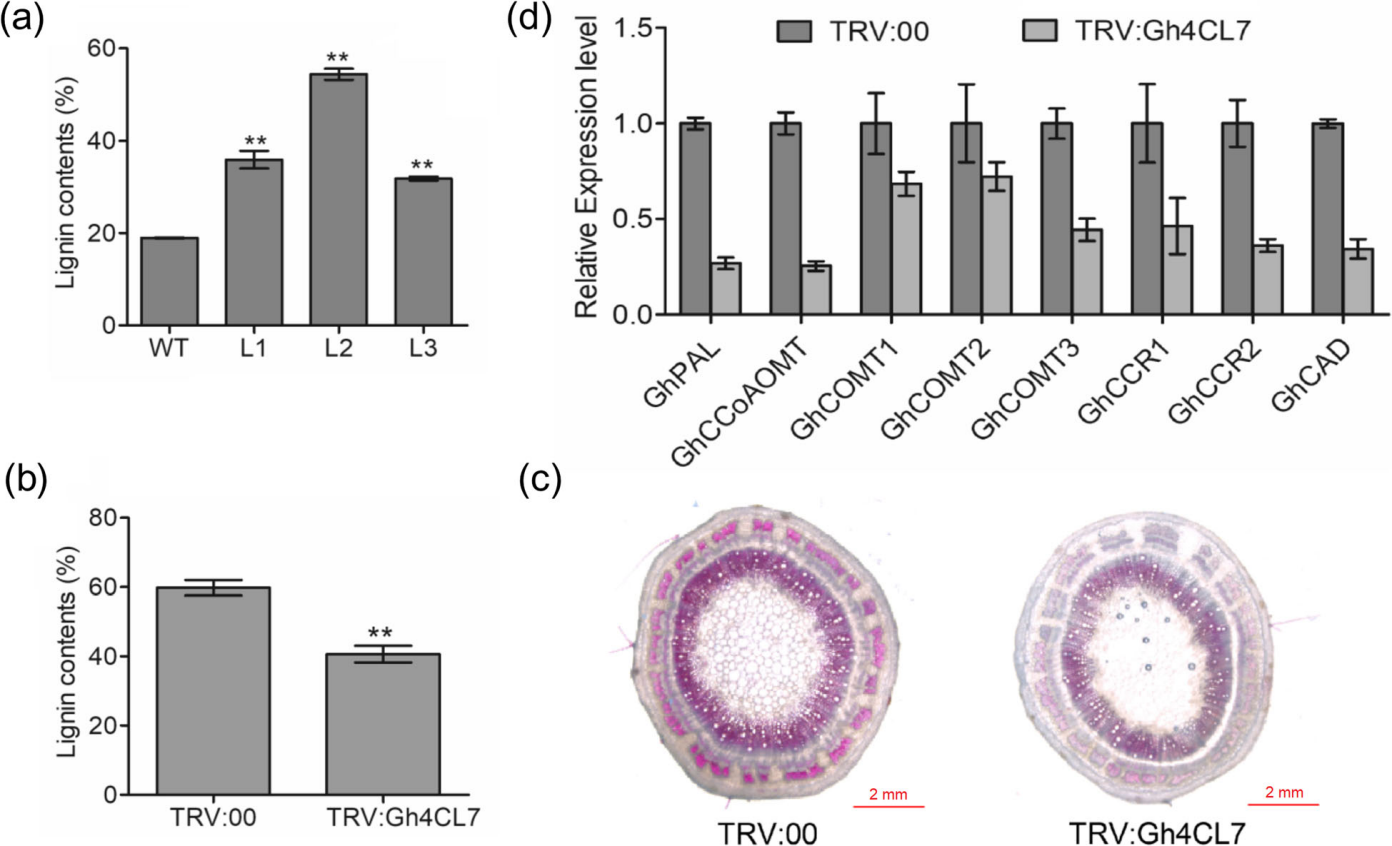
Analyses of lignin contents and the expression level of genes related to lignin biosynthesis. **a**-**b** Comparison of lignin contents in *Gh4CL7*-OE *Arabidopsis* plants (**a**) and in *Gh4CL7*-silencing cotton plants (**b**). **c** Stem sections from the *TRV:Gh4CL7* and *TRV:00* cotton plants stained with phloroglucinol-HCl. **d** The relative expression levels of genes related to lignin biosynthesis. Data were represented as the mean ± SE of three biological replicates; asterisks indicate levels of significance based on t-test (* *P* < 0.05, ** *P* < 0.01)

We used VIGS to silence the expression of *Gh4CL7* using the TRV vector (*TRV:Gh4CL7*; Additional file 1: Figure S4). *TRV:GhCHLI* was used as a positive control of the VIGS experiment (Additional file 1: Figure S5). *Arabidopsis thaliana* plants overexpressing *Gh4CL7* (*Gh4CL7*-OE) were obtained by the floral dip method. *Gh4CL7* belongs to class I whose genes have been shown to regulate lignin biosynthesis [10, 22]. We thus first investigated whether or not *Gh4CL7* is also involved in lignin biosynthesis by comparing the lignin contents of the *Gh4CL7-*OE *Arabidopsis* lines and *TRV:Gh4CL7* cotton plants with that of their corresponding control plants. The lignin content increased by approximately 10% in the *Gh4CL7*-OE lines compared with wild-type (WT) plants (Fig. 4a), while decreased by approximately 20% in the *TRV:Gh4CL7* plants compared with the *TRV:00* plants (Fig. 4b). Additionally, the stem of the *TRV:Gh4CL7* plants were sectioned and stained with phloroglucinol-HCl to detect the presence of lignin (Fig. 4c). We found that the stem section of the *TRV:Gh4CL7* plants with reduced lignin content exhibited light red color, but the *TRV:00* plants displayed typically purple-red color after phloroglucinol-HCl staining. These results suggested that *Gh4CL7* is related to lignin synthesis. We also analysed the expression level of the phenylpropane pathway genes that are related to lignin biosynthesis, including *GhPAL*, *GhCCoAOMT1*, *GhCOMT1*, *GhCOMT2*, *GhCOMT3*, *GhCCR1*, *GhCCR2*, and *GhCAD*. The relative expression level of these genes were lower in the *TRV:Gh4CL7* plants than in *TRV:00* (Fig. 4d), indicating that *Gh4CL7* could affect the accumulation of lignin by regulating the transcription level of these downstream genes of the lignin biosynthesis pathway.

### Silencing of *Gh4CL7* compromises tolerance of cotton to drought stress

**Fig. 5:**
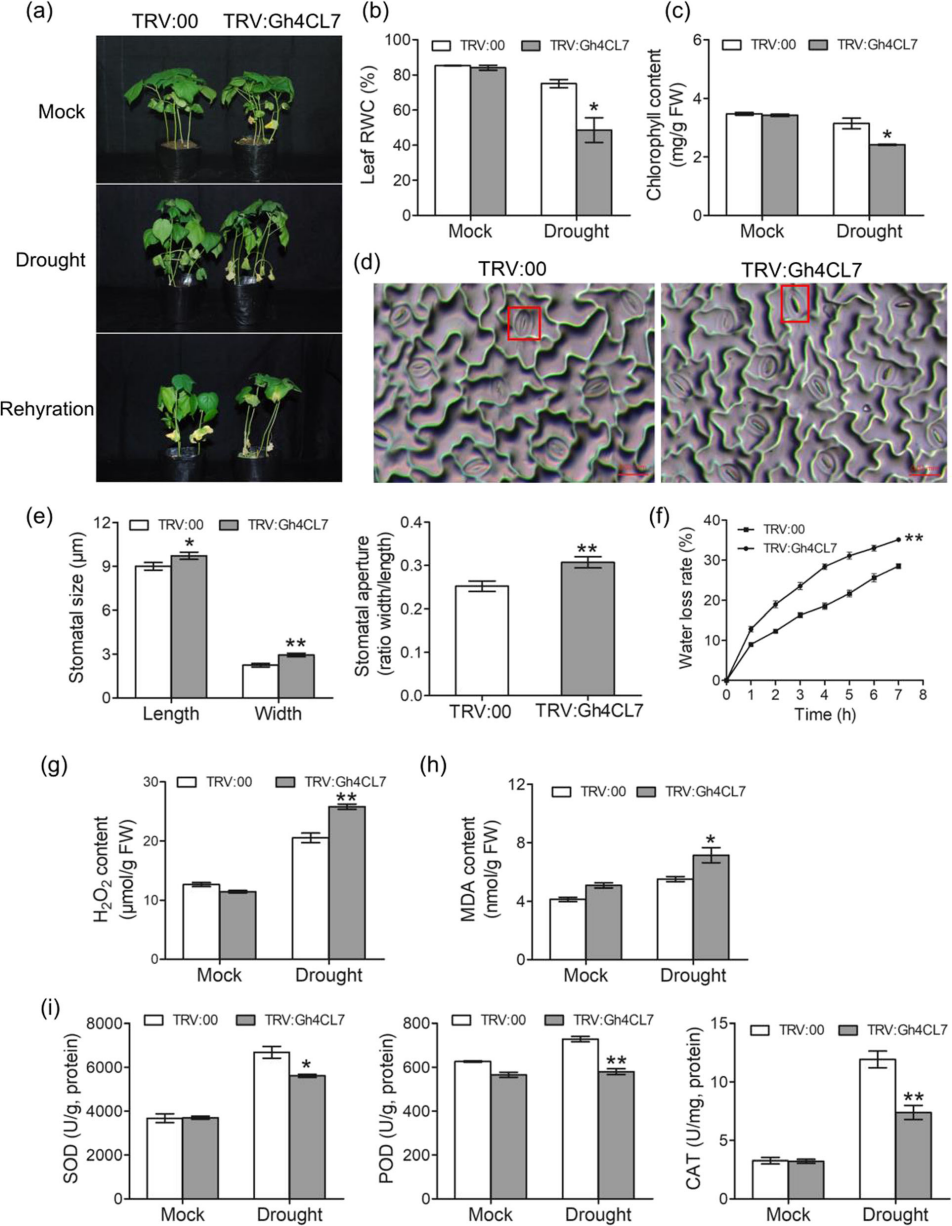
Drought tolerance analysis of the *Gh4CL7*-silencing cotton plants. **a** Representative phenotypes of the *TRV:00* (CK) and *TRV:Gh4CL7* (VIGS) plants after 3 weeks of drought treatment. **b-c** Leaf RWC and chlorophyll contents of CK and VIGS plants under drought stress. **d-e** Comparison of stomata in the CK and VIGS plants. **f** Water loss rate of detached leaves from the CK and VIGS plants. **g-h** Comparison of H_2_O_2_ and MDA contents in the CK and VIGS plants. **i** Activities of antioxidative enzymes in the CK and VIGS plants. Mock, normal growth conditions; drought, 3 weeks of water deficit conditions. Statistical significance was determined by the student test. * and ** represent significant at *P* < 0.05 and *P* < 0.01, respectively

Phenotypic difference between the *TRV:Gh4CL7* and *TRV:00* plants was observed after 20 days of water deficiency treatment. Compared to the *TRV:00* plants, the *TRV:Gh4CL7* plants displayed severe wilting and yellowing leaves (Fig. 5a), consistent with a lower leaf relative water content (RWC) (Fig. 5b) and a decrease chlorophyll contents (Fig. 5c). Besides, it was also found that the size and the ratio of width to length of stomata significantly increased in the *TRV:Gh4CL7* plants (Fig. 5d-f), which might accelerate the transpiration rate under drought conditions, consistent with the observed higher water loss relative (WLR) (Fig. 5g). The hydrogen peroxide (H_2_O_2_) content and malondialdehyde (MDA) level were measured to reflect the cell damage or injury in *TRV:Gh4CL7* and *TRV:00* plants. During drought stress, the *TRV:Gh4CL7* plants accumulated more MDA (Fig. 5h) and H_2_O_2_ (Fig. 5i) compared to the *TRV:00* plants. The activities of superoxide dismutase (SOD), peroxidase (POD) and catalase (CAT) in the *TRV:Gh4CL7* and *TRV:00* plants were also measured to explore the function of *Gh4CL7* in the modulation of antioxidant enzymes (Fig. 5j). As expected, under drought stress conditions, the *TRV:Gh4CL7* plants displayed a significantly reduced activity of SOD, POD and CAT as compared to the *TRV:00* plants. Additionally, six stress-related genes (*GhABI4*, *GhABF4*, *GhLEA14*, *GhRD22*, *GhRD29* and *GhNCED1*) were down-regulated in the *TRV:Gh4CL7* plants after drought treatment (Additional file 1: Figure S6). These results suggested that silencing of *Gh4CL7* decreases tolerance of cotton to drought stress.

### Overexpression of *Gh4CL7* in *Arabidopsis* enhances drought tolerance

**Fig. 6:**
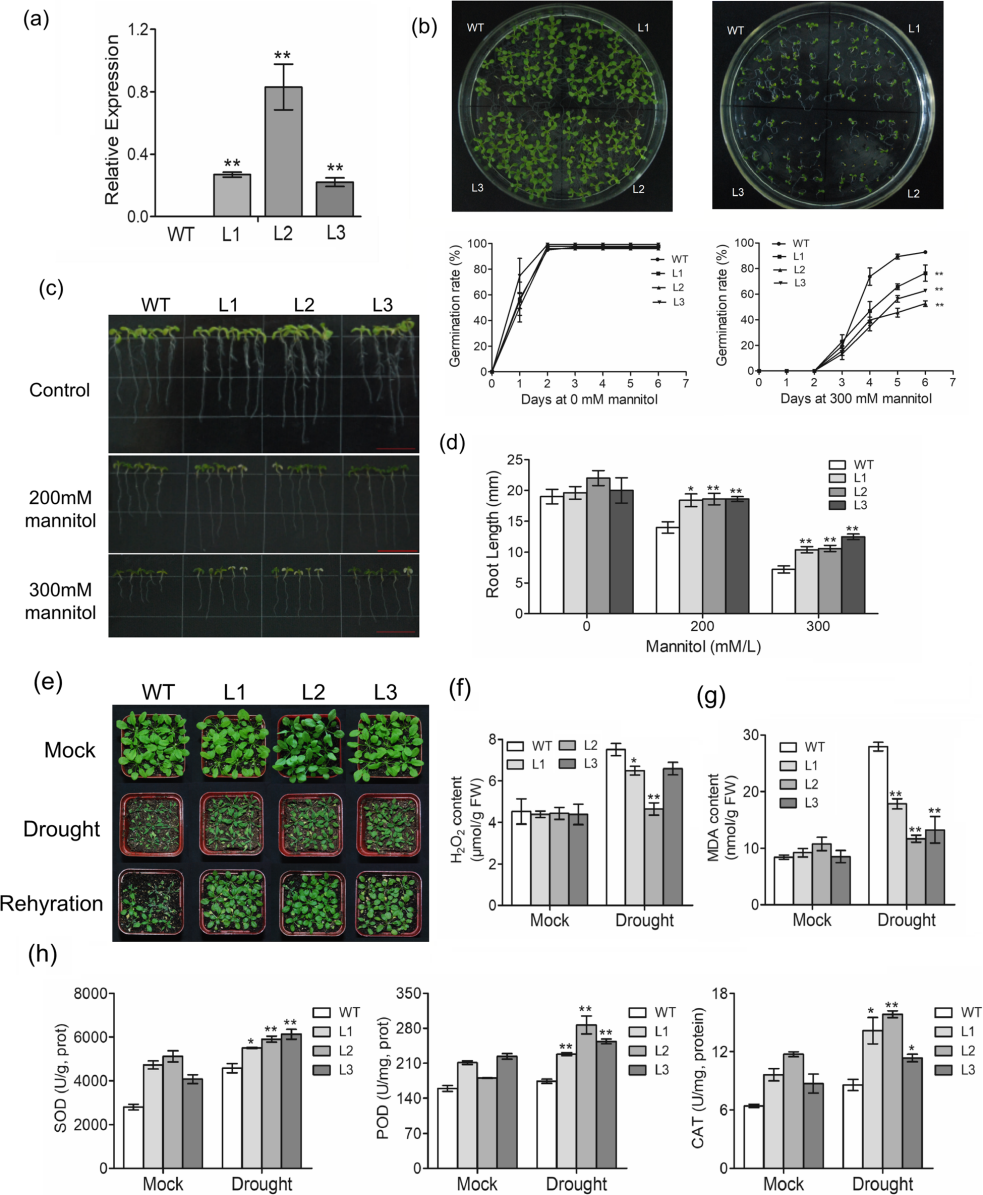
Drought tolerance analysis of the *Gh4CL7*-overexpressing *Arabidopsis* plants. **a** The relative expression level of *Gh4CL7* in three independent *Gh4CL7*-OE *Arabidopsis* plants. **b** Germination rate of the *Gh4CL7*-OE *Arabidopsis* seeds on 1/2 MS supplemented with 0 and 300 mM mannitol. **c-d** Root elongation of the 6-days-old *Gh4CL7*-OE *Arabidopsis* seedlings on 1/2 MS supplemented with 0, 200, and 300 mM mannitol. Bar = 1 cm. **e** Phenotypic assay of the *Gh4CL7*-OE and WT *Arabidopsis* plants during drought treatment. **f-h** The contents of H_2_O_2_ and MDA and antioxidative enzyme activity in *Gh4CL7*-OE and WT plants under normal and water deficit conditions. Ten-days-old seedlings were transplanted to soil and regularly watered for 2 weeks. For drought treatment, irrigation was terminated for 2 weeks. For the rehydration treatment, the plants were re-watered 3 days after the drought treatment

**Fig. 7:**
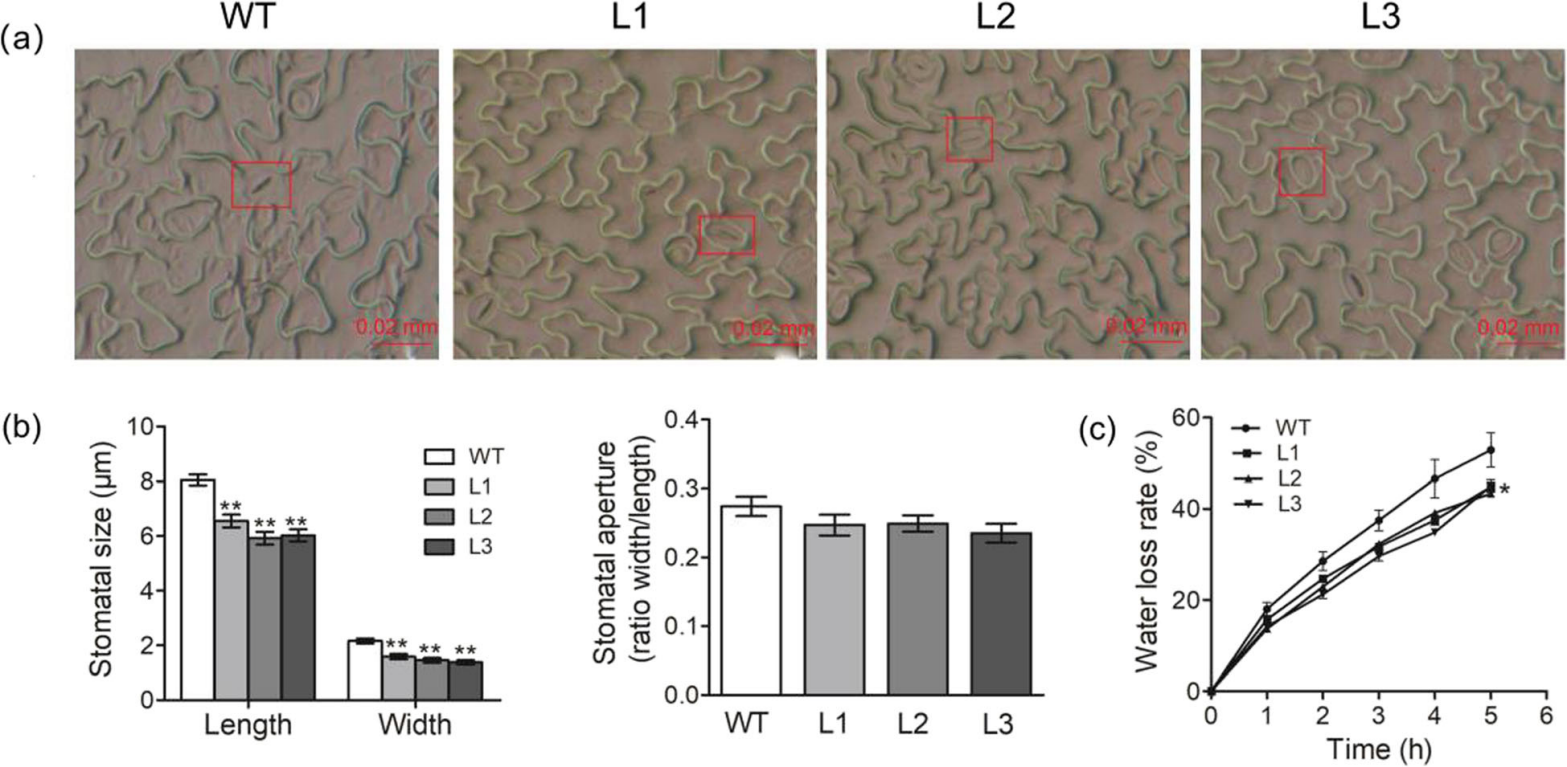
Stomatal opening in the WT and *Gh4CL7*-OE *Arabidopsis* plants under drought stress. **a-b** Comparison of stomata in the WT and *Gh4CL7*-OE plants after 14 days of water-withholding stress. **c** WLR of detached leaves from WT and *Gh4CL7*-OE *Arabidopsis* plants. Data were represented as the mean ± SE of three biological replicates. Statistical significance was determined by the student test. * and ** represent significant at *P* < 0.05 and *P* < 0.01, respectively

We further investigated the function of *Gh4CL7* in response to drought stress using *Arabidopsis* plants overexpressing *Gh4CL7*. Three independent *Gh4CL7-*OE lines that showed an elevated level of *Gh4CL7* (Fig. 6a) were selected for phenotyping under the drought stress conditions. Compared to the WT plants, the three *Gh4CL7-*OE lines had a decreased germination rate (Fig. 6b), but showed a significantly increased root length under the mannitol stress conditions (Fig. 6c, d). Three-weeks-old seedlings of *Gh4CL7-*OE and WT were used for water deficiency treatment. No obvious phenotypic difference was observed between *Gh4CL7-*OE and WT by the mock treatment. However, the *Gh4CL7-*OE plants showed much less damage than WT after 10 days of water deficiency (Fig. 6e). Under drought stress conditions, the H_2_O_2_ content and MDA level in the *Gh4CL7-*OE plants were relatively lower than that in WT (Fig. 6f-g), but the SOD, POD and CAT activities were significantly higher (Fig. 6h). Additionally, the size and the ratio of width to length of stomata significantly decreased in the *Gh4CL7*-OE *Arabidopsis* plants (Fig. 7a-b), consistent with a lower WLR observed in those plants (Fig. 7c)*.* To further elucidate the possible mechanism of *Gh4CL7* in response to drought stress, the transcript levels of four known ABA-responsive genes (*AtRD29B*, *AtRD22*, *AtABI4*, *AtCOR15A*) and two ABA-biosynthesis genes (*AtNCED3* and *AtNCED5*) were analyzed in the *Gh4CL7-*OE lines and WT plants after drought stress treatment. The qRT-PCR data showed that the expression levels of these genes were induced in *Gh4CL7-*OE, but not or just slightly induced in WT by drought stress (Additional file 1: Figure S7). These results indicated that overexpression of *Gh4CL7* could enhance the tolerance of transgenic *Arabidopsis* plants to drought stress.

## Discussion

The *4CL* gene family has been characterized in several plants, including *Arabidopsis thaliana, Populus trichocarpa, Oryza sativa* and *Glycine max* [11, 23–25]. Genes of this family have been reported to function not only in plant growth and development [16, 22, 26], but also in response to biotic and abiotic stresses [7, 27]. However, no comprehensive analysis of the *4CL* genes has been documented in *G. hirsutum*. In this study, we did genome-wide identification of *4CL* genes in *G. hirsutum* and investigated their expression profiles in various tissues and under different stress conditions with an aim to identify *4CL* gene(s) with a potential role in stress tolerance. In total, 34 *Gh4CL* members were identified in the *G. hirsutum* genome (Table 1). In other plant species, such as *A. thaliana*, *4CL* genes were divided into three classes, i.e. class I, class II and *4CL-like* [13]. The 34 *Gh4CL* genes could also be clustered into three classes. We named the *4CL-like* class as class III, which contains the largest number of *Gh4CL* genes (in total 25) together with *At4CL6–9*, *At4CL11*, and *Ii4CL1* (Fig. 1b). The class III *Gh4CLs* cannot catalyze any of the hydroxycinnamic acid substrates into the corresponding CoA esters, their function is different from that of class I (related to the lignin biosynthesis) and class II (related to the biosynthesis of flavonoids) *4CL* genes [13, 18, 28]. Multiple sequence alignments revealed that all 4CL-like proteins contained similar structural components, e.g. conserved Box I and Box II domains without known specific biochemical function (Additional file 1: Figure S2) [29]. Gene structure analysis showed that *Gh4CL* genes of the same class share a similar intron-exon structure (Fig. 2b), similar to the observations made in other gene families [30–32].

*Cis*-elements located in the promoter region of genes play key roles in the developmental and environmental regulation of gene expression [33]. According to *cis*-element analysis, the promoter regions of *Gh4CL* genes possess elements related to stress responses, such as ABRE, TC-rich, LTR, MBS, TGA-element, TCA-element, CGTCA-motif and TGACG-motif [34–36], suggesting a potential role of the *Gh4CL* genes with these *cis*-element(s) in regulationof stresses, such as drought, salt, heat, ABA, and low temperature. *4CL* genes have been shown to be involved in response to stresses in other plants [14, 27]. Based on transcriptome data, 26 *Gh4CL* genes were differentially expressed between the stress treatment and the mock, and many *Gh4CL* genes were induced by drought, including *Gh4CL7* (Fig. 3a). The promoter of *Gh4CL7* contains an MBS *cis*-acting element, which may be associated with up-regulation of *Gh4CL7* upon drought treatment (Fig. 3b). In addition, the *Gh4CL* genes showed different expression profiles in different tissues (Fig. 2), suggesting that they perhaps play a broad range of roles in cotton growth and development.

Abiotic stresses often disrupt the balance between reactive oxygen species (ROS) production and clearance in cells, leading to increased ROS concentrations and oxidative damage to biofilms, proteins, DNA, and RNA, thereby inhibiting plant growth and development [37, 38]. Therefore, scavenging ROS is essential for plants to resist abiotic stresses. H_2_O_2_ is one of the ROS, and its over accumulation in the plant cell can cause oxidative damage, while a low level of H_2_O_2_ concentration correlates with drought tolerance [39]. In the *Gh4CL7* gene-silenced plants, the H_2_O_2_ content was found to be increased significantly under drought stress, so was the MDA level, an indicator of ROS destructive effects [40]. SOD, POD, and CAT are antioxidant enzymes in plant cells which scavenge the toxic ROS and lead to enhanced tolerance under stress conditions [41]. We found that the activities of SOD, POD, and CAT were lower in the *TRV:Gh4CL7* plants than in the *TRV:00* plants indicating that the reduced ability of the *Gh4CL7* silenced plants to scavenge ROS that might have led to membrane damages and chlorophyll content reduction. On the other hand, the *Arabodopsis* plants overexpressing *Gh4CL7* had a lower level of H_2_O_2_ and MDA and a higher activity of antioxidant enzymes (SOD, POD, and CAT) compared to WT under the drought stress conditions. These results are consistent with previous finding that *Fm4CL* had a role in drought tolerance by modulating the level of ROS [19].

Drought stress affects crop yield and quality through its negative influence on seed germination, seedling growth, photosynthesis, and transpiration [1]. We found that *A. thaliana* plants overexpressing *Gh4CL7* had longer roots but a lower germination rate than WT plants under the osmotic stress conditions, suggesting that *Gh4CL7* played a negative role in seed germination, but a positive role in promoting root elongation under the osmotic stress conditions. Breeding crops with thriving and deeper root systems is the goal of geneticists and breeders, because it can increase productivity of crops under drought conditions [42]. Longer roots might be a result of changed ABA signaling pathway, which plays a crucial role in root development under drought stress [43, 44], as well as many other factors related to drought responses, including stomata closure and stress-gene regulation [45, 46]. Under drought stress conditions, the closed stomata can decrease transpiration rate that helps plants to resist adverse environmental conditions. Our results showed that the size of stomatas was bigger in the *TRV:Gh4CL7* plants, suggesting a positive role of *Gh4CL7* in reducing transpiration rate that allows cotton to maintain a more favourable water balance, and effectively improves drought tolerance. This was supported by the observation of a higher WLR in leaves of the *TRV:Gh4CL7* plants than those of WT plants.

Lignin is the second largest polymer in plants after cellulose [47]. It provides mechanical support to plants by increasing cell wall hardness and enhancing compressive strength of cells [48, 49]. We found that repressing the expression level of *Gh4CL7* in *G. hirsutum* reduced the lignin content and led to a reduction in drought resistance, consistent with the result of rice plants with a decreased lignin content being more prone to drought stress [50]. Studies in *Fraxinus mandshurica* also showed that decreased lignin content resulted in drought resistance reduction [19]. The hydrophobicity of lignin is thought to have an inhibitory effect on the transpiration of plant tissue under drought conditions [51], that could be the reason for *Arabidopsis* plants overexpressing *Gh4CL7* with an increased level of lignin content being more resistant to drought.

## Conclusions

The findings of this study demonstrate that the *Gh4CL7*-silencing cotton plants had an increased sensitivity of drought stress while overexpressing *Gh4CL7* enhanced tolerance of drought stress in *Arabidopsis. Gh4CL7* conferred tolerance to drought stress by increasing lignin content, improving the antioxidant system, closing stomata, and up-regulating the transcription levels of ABA-responsive genes. Although the exact mechanism of *Gh4CL7*-mediated drought tolerance is still yet to be uncovered, our results provide evidence for the role of *Gh4CL7* in combating drought stress.

## Methods

### Identification of the *4CL* family genes in *Gossypium hirsutum*

The annotated protein sequences of *G. hirsutum* [21] were downloaded from CottonGen (https://www.cottongen.org/). The hidden Markov model file corresponding to the AMP-binding domain (PF00501) was downloaded from the Pfam protein family database (http://pfam.xfam.org/) and used as query (*P* < 0.001) [52] to search for the *4CL* genes in *G. hirsutum* with HMMER 3.0 [53]. The existence of the AMP-binding domain sequences was examined using the Pfam, SMART (http://smart.embl-heidelberg.de/), and National Center for Biotechnology Information (NCBI) Conserved Domains (http://www.ncbi.nlm.nih.gov/Structure/cdd/wrpsb.cgi) databases [54, 55].

### Gene structure, conserved motif and promoter analyses

The length, molecular weight (MW), and isoelectric point (*p*I) of the identified Gh4CL proteins were calculated using the ExPasy website tools (http://web.expasy.org/protparam/) [56]. PSORT software (https://psort.hgc.jp/) was used for predicting subcellular localization [57]. Gene Structure Display Server 2.0 (GSDS, http://gsds.cbi.pku.edu.cn/) was used for intron and exon analysis [58]. The conserved motifs in the Gh4CL protein sequences were identified using the Multiple Expectation Maximization for Motif Elicitation (MEME) program (version 5.0.5, http://meme-suite.org/tools/meme) [59]. The potential *cis*-elements in the promoter sequences (up to 2000-bp upstream ATG) of *Gh4CL* genes were identified using the PlantCARE program (http://bioinformatics.psb.ugent.be/webtools/plantcare/html/).

### Phylogenetic tree, chromosomal distribution and syntenic relationship analyses

The multiple sequence alignment of Gh4CLs was done by Clustal X [60] and DNAMAN (version 5.2.2). The 4CL homologous protein sequences of *Arabidopsis thaliana* (At4CL1: OAP14948; At4CL2: OAP07084; At4CL3: AEE34324; At4CL4: AY376731; At4CL5: AY250839; At4CL7: AY376733; At4CL9: AF360250 At4CL11: AY376735), *Glycine max* (Gm4CL1: AF279267; Gm4CL2: AF002259; Gm4CL3: AF002258; Gm4CL4: X69955), *Rubus idaeus* (Ri4CL1: AF239687; Ri4CL2: AF239686; Ri4CL3: AF239685), *Populus tremuloides* (Pt4CL1: U12012; Pt4CL2: U12013), and *Isatis tinctoria* (Ii4CL1: ADG46006; Ii4CL2: KC430622; Ii4CL3: KC430623) were downloaded from the NCBI (http://www.ncbi.nlm.nih.gov/, accessed on 7 May 2018) and used for the phylogenetic tree analysis by using the neighbor joining method (NJ) in MEGA 6.0 [61] with 1000 repetitions for the bootstrap test.

All the *Gh4CL* genes were mapped to *G. hirsutum* chromosomes, based on their physical location information, using TBtools [62]. Two or more homologous/paralogous *4CL* genes located at a chromosomal region of < 200 kb were considered to be generated by tandem duplication events [63]. Multiple Collinearity Scan toolkit (MCScanX) was used to analyze the gene duplication events with the default parameters [64]. Non-synonymous (Ka) and synonymous (Ks) substitutions in each paralogous *Gh4CL* gene pair were calculated using KaKs_Calculator 2.0 [65].

### Vector construction and genetic transformation

To generate *Arabidopsis* overexpressing lines, the coding sequence of *Gh4CL7* was amplified using PrimeSTAR DNA polymerase (TaKaRa, Tokyo, Japan) with the gene-specific forward and reverse primers and ligated into the pCAMBIA2300 vector driven by the CaMV35S promoter. The expression vector pCAMBIA2300-*Gh4CL7* was transformed into *Agrobacterium tumefaciens* strain GV3101. *Arabidopsis* ecotype Col-0 (wild-type, provided by Microbiology Institute of Chinese Academy of Sciences, Beijing, China) was used in genetic transformation by the floral dip method [66]. The harvested T0 generation seeds were selected on 1/2 Murashige and Skoog (MS) medium with 50 mg/L kanamycin, and the resistant plants were further validated by PCR. Single-copy lines with a segregation ratio of 3:1 were selected and planted until T3 generation. The transgenic *Arabidopsis* OE lines were grown in a growth chamber with 16 h light/8 h dark scheme, and the growth temperature was set at around 23 °C [67].

The pTRV1 and pTRV2 VIGS vectors were kindly provided by Prof. Longfu Zhu of Huazhong Agricultural University. A 287-bp fragment from the coding sequence of *Gh4CL7* was amplified from cDNA of *G. hirsutum* cultivar stem and subsequently cloned into pTRV2 using *Bam*H1 and *Kpn*1 double digestions to generate the *TRV:Gh4CL7* vector. After PCR and double digestion confirmation, the *TRV:Gh4CL7* construct was transformed into *A. tumefaciens* strain GV3101 by electroporation. *G. hirsutum* cv. Junmian-1 (provided by the Cotton Research Institute of Shihezi University, Shihezi, Xinjiang Province, China) was used in VIGS and was grown in pots filled with soil mix (3:1, humus:vermiculite) and placed in growth room at 24 ± 1 °C with 16 h light/8 h dark (200 μmol/m^2^/s photon flux density). VIGS was done as previously described by Xiong and colleagues [67].

### Drought tolerance assays

PEG and natural drought treatments were conducted to investigate the function of *Gh4CL7* in response to osmotic and drought stresses. For the PEG treatments, *TRV:Gh4CL7* (VIGS) and *TRV:00* (control) plants at three-leaf stage were subjected to stress by 20% PEG6000 (w/v). Leaf samples were collected at 0 (ck), 1, 3, 6, 9, 12, 24 h after treatment for RNA extraction. For the natural drought treatments, *TRV:Gh4CL7* and *TRV:00* plants were not irrigated for 3 weeks, followed by re-watering once. After 15 days of water deprivation, plant leaves were collected for RNA extraction and determination of physiological parameters.

For analyses of seed germination and root elongation, seeds or 3-day-old seedlings of *Gh4CL7 -*OE *Arabidopsis* lines and WT were grown on 1/2 MS plates supplemented with 0 (control), 200, 300 mM mannitol for 6 days. In the vegetative growth stage, 7 days-old transgenic *Arabidopsis* and WT plants were transplanted into soil and watered once every 3 days for 2 weeks, then kept without irrigation for 2 weeks, followed by re-watering once. After 10 days of drought treatment, the rosette leaves were collected for RNA extraction and determination of physiological parameters.

### Determination of drought stress-related physiological parameters

The thiobarbituric acid (TBA) colorimetric method was used to measure the content of MDA according to the instruction of the malondialdehyde quantification kit (Suzhou Comin Biochemistry Co. Ltd., Su Zhou, China). H_2_O_2_ concentrations were determined by using the H_2_O_2_ determination kit (Suzhou Comin Biochemistry Co. Ltd., Su Zhou, China) by following the manufacturer’s instructions. Measurement of the activities of antioxidative enzymes was performed using 0.1 g leaf sample according to the instructions of the POD, SOD and CAT Assay Kit (Suzhou Comin Biochemistry Co. Ltd., Su Zhou, China). Total contents of chlorophyll were calculated according to the method described by Porra and colleagues [68]. Absorbances at different wavelengths were measured by the U-5100 UV/VIS spectrophotometer (HITACHI, Tokyo, Japan).

### Measurements of WLR, RWC and stomatal aperture

For water loss assays, leaves from *G. hirsutum* and *Arabidopsis* were immediately weighed and placed in a growth room at room temperature with a humidity level of about 60%. The leaves were weighed once per hour. WLR was estimated as the percentage of fresh weight lost relative to the initial fresh weight [69]. For measurement of the RWC, fresh leaves were detached from plants and their fresh weights (FW) were immediately recorded. Then, the leaves were placed in distilled water for 8 h at 25 °C in the dark and measured the turgid weight (TW). Dry weights (DW) were recorded by drying samples at 65 °C until constant weight. RWC was calculated as (FW - DW)/(TW - DW) × 100 [70].

Stomatal pore area and size were determined using the rapid imprinting technique [71]. The abaxial leaf surfaces were covered with transparent nail polish and air dried at room temperature. The nail polish imprints were made into temporary slices and photographed by Zeiss microscope (SteREO Discovery.V20, Germany) with 300× magnifications. The length and width of stomatal pores were measured using the Image J software and the relative aperture area was calculated based on the ratio of width to length.

### Lignin content measurement and histochemical staining

Lignin content was determined by the acetyl bromide method [72] and the phloroglucinol-HCl color-developing method. Transections of stem (above the cotyledons) of the *Gh4CL7*-silencing cotton plants at 28 days after sowing were made by hand cutting using razor blades. The transverse sections were immersed in 1 mL of 1 M hydrochloric acid for 3 min, followed by transferring into 1 ml 10% phloroglucinol ethanol-hydrochloric solution for 1 min, and visualized immediately under the microscope (Zeiss, SteREO Discovery.V20, Germany) for photographing [73].

### RNA extraction and quantitative real-time PCR analysis

To investigate gene expression patterns, total RNA was extracted from leaves of *G. hirsutum* and *Arabidopsis* with the EASYspin Plus plant RNA kit (Aidlab, Beijing, China). RNA was reverse transcribed into cDNA using the M-mlv reverse transcript system (TAKARA, Da Lian, China). The qRT-PCR was performed using the Power SYBR Green PCR Master Mixture (Roche, Rotkreuz, Switzerland) on a Light Cycler® 480 II system (Roche, Rotkreuz, Switzerland) under the following conditions: initial pre-incubation at 95 °C for 5 min, followed by 40 cycles at 94 °C for 10 s, 59 °C for 10 s, and 72 °C for 10 s. The relative expression level of genes was analyzed by the 2^-△△^Ct method. The results were presented as the mean of three biological replications. The *G. hirsutum histone3* gene and *Arabidopsis EF-lα* gene were used as the reference genes. All the primers used in this study were designed using the NCBI primer designing tool (https://www.ncbi.nlm.nih.gov/tools/primer-blast/, accessed on 27 August 2018) and listed in Additional file 2: Table S3.

### Statistical analyses

Statistical analyses and data plotting were performed using SPSS and Graphpad Prism 5, respectively. ** and * represent significant differences at *P* < 0.01 and *P* < 0.05, respectively.

## Supplementary information

**Additional file 1: Figure S1.** Gene structure and motif pattern of the *Gh4CL* genes. **a** Structural analysis. The left panel shows the neighbor-joining phylogenetic tree based on the amino acid sequences of the *Gh4CLs*. The classes I, II and III were marked correspondingly. The right panel shows the exon-intron structure of each *Gh4CL* gene with exons showing in orange boxes, introns in black lines between exons, and upstream/downstream UTRs in blue boxes. The number indicates the phase of the corresponding introns. The length of the *Gh4CL* genes is indicted by the scale line at the bottom. **b** Motif analysis. The motif analysis was performed by the MEME suite. Twenty motifs were detected and are displayed in color coded boxes. The length of proteins is indicted by the scale line at the bottom. **Figure S2.** Alignment of multiple Gh4CL and selected At4CL domain amino acid sequences. Multiple sequence alignment was performed using Clustal X. Box I and Box II represent the two conserved domains of the Gh4CL proteins. **Figure S3.** Analysis of *cis*-elements in the promoter of the *Gh4CL* genes. The numbers of different *cis*-elements are presented in the form of bar graphs. **Figure S4.** Relative expression levels of *Gh4CL7* in plants infiltrated with *TRV:00* and *TRV:Gh4CL7* (*n* = 5). Total RNA was extracted from leaves at 2 weeks post-infiltration. Transcript levels were determined by qRT-PCR using *GhUBQ7* as control. **Figure S5.** Silencing of the endogenous magnesium chelatase subunit I gene (*GhCHLI*) in cotton through VIGS. The leaf bleaching phenotype was observed 2 weeks after infiltration in TRV: GhCHLI plants. **Figure S6.** Transcription levels of stress responsive genes in CK and *Gh4CL7*-siliencing cotton plants. The normal condition plants were used as controls. *GhUBQ7* gene was used as an internal control. All the gene expressions were normalized to the corresponding transcript levels in CK plants at normal condition. All the data represent mean ± SE for three biological replications. **Figure S7.** Transcription levels of stress responsive genes in transgenic and WT *Arabidopsis* plants. The normal condition plants were used as controls. *AtEF-Lα* gene was used as an internal control. All the gene expressions were normalized to the corresponding transcript levels in WT plants at normal condition. All the data represent mean ± SE for three biological replications.

**Additional file 2: Table S1.** Ka, Ks, Ka/Ks values for the Gh4CL paralogous gene pairs. **Table S2.** Information of the 20 motifs of Gh4CL proteins. **Table S3.** The primers used in qRT-PCR analysis.

## Data Availability

The sequencing data are available in the NCBI Sequence Read Archive (SRA) database under the accession number PRJNA248163. All other data generated or analyzed during this study are included in this manuscript.

## Abbreviations

4CL: 4-coumarate-CoA ligases
VIGS: Virus-induced gene silencing
OE: Overexpression
RWC: Relative water content
MDA: Malondialdehyde
H_2_O_2_: Hydrogen peroxide
WLR: Water loss relative
PEG: Polyethylene glycol
DPA: Days post anthesis
qRT-PCR: Quantitative reverse transcription polymerase chain reaction
CAT: Catalase
POD: Peroxidase
SOD: Superoxide dismutase
ROS: Reactive oxygen species
WT: Wild type
TRV:Gh4CL7: *Gh4CL7* VIGS plants
TRV:00: Empty vector VIGS plants
FPKM: Fragments per kilobase of transcript per million mapped reads
