# Correction to: Characterization of the *Gh4CL* gene family reveals a role of *Gh4CL7* in drought tolerance

**DOI:** 10.1186/s12870-021-02834-9

**Published:** 2021-01-28

**Authors:** Shi-Chao Sun, Xian-Peng Xiong, Xiao-Li Zhang, Hong-Jie Feng, Qian-Hao Zhu, Jie Sun, Yan-Jun Li

**Affiliations:** 1 Key Laboratory of Oasis Eco-agriculture, College of Agriculture, Shihezi University, Shihezi, 832000 Xinjiang China; 2 Key Laboratory of Cotton Biology, Institute of Cotton Research, Chinese Academy of Agricultural Sciences, Anyang, 455000 Henan China; 3 CSIRO Agriculture and Food, GPO Box 1700, Canberra, 2601 Australia

**Correction to: BMC Plant Biol (2020) 20:125**

**https://doi.org/10.1186/s12870-020-2329-2**

In the original publication [1] there was an incorrect funding number. The incorrect and correct funding number are published in this correction article. The original article has been updated.

**Incorrect funding**

- This work was supported by the National Natural Science Foundation of China (Grant No: **31906438**, 31360347), the National Key Research and Development Program of China (Grant No: 2016YFD0100200) and the Breeding Program of Shihezi University (Grant No:YZZX201601). The funding bodies had no role in the design of the study and collection, analysis, and interpretation of data and in writing the manuscript.

**Correct funding**

- This work was supported by the National Natural Science Foundation of China (Grant No: **31960438**, 31360347), the National Key Research and Development Program of China (Grant No: 2016YFD0100200) and the Breeding Program of Shihezi University (Grant No:YZZX201601). The funding bodies had no role in the design of the study and collection, analysis, and interpretation of data and in writing the manuscript.

## References

[1] Sun SC, Xiong XP, Zhang XL (2020). Characterization of the *Gh4CL* gene family reveals a role of *Gh4CL7* in drought tolerance. BMC Plant Biol.

