# Supplementary figures and images for: Characterization of the Gh4CL gene family reveals a role of Gh4CL7 in drought tolerance
Source: BMC Plant Biol. 2020 Mar 23;20:125. doi: 10.1186/s12870-020-2329-2 (PMC7092558; doi:10.1186/s12870-020-2329-2)

Fig. S1


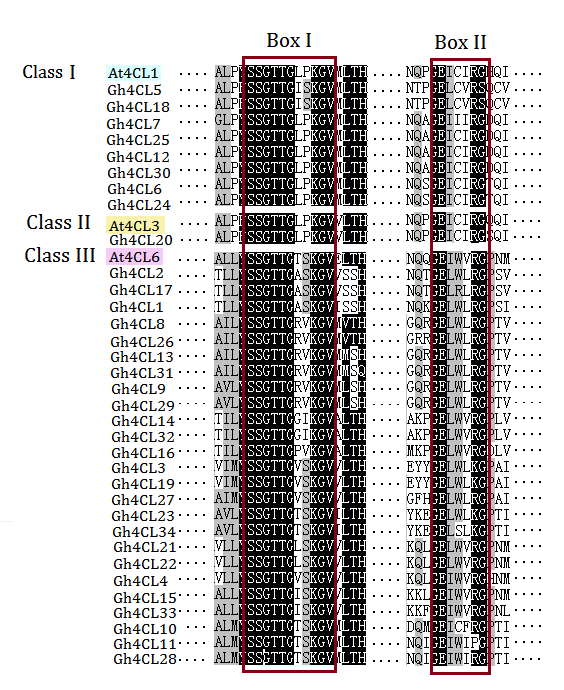


Fig. S2


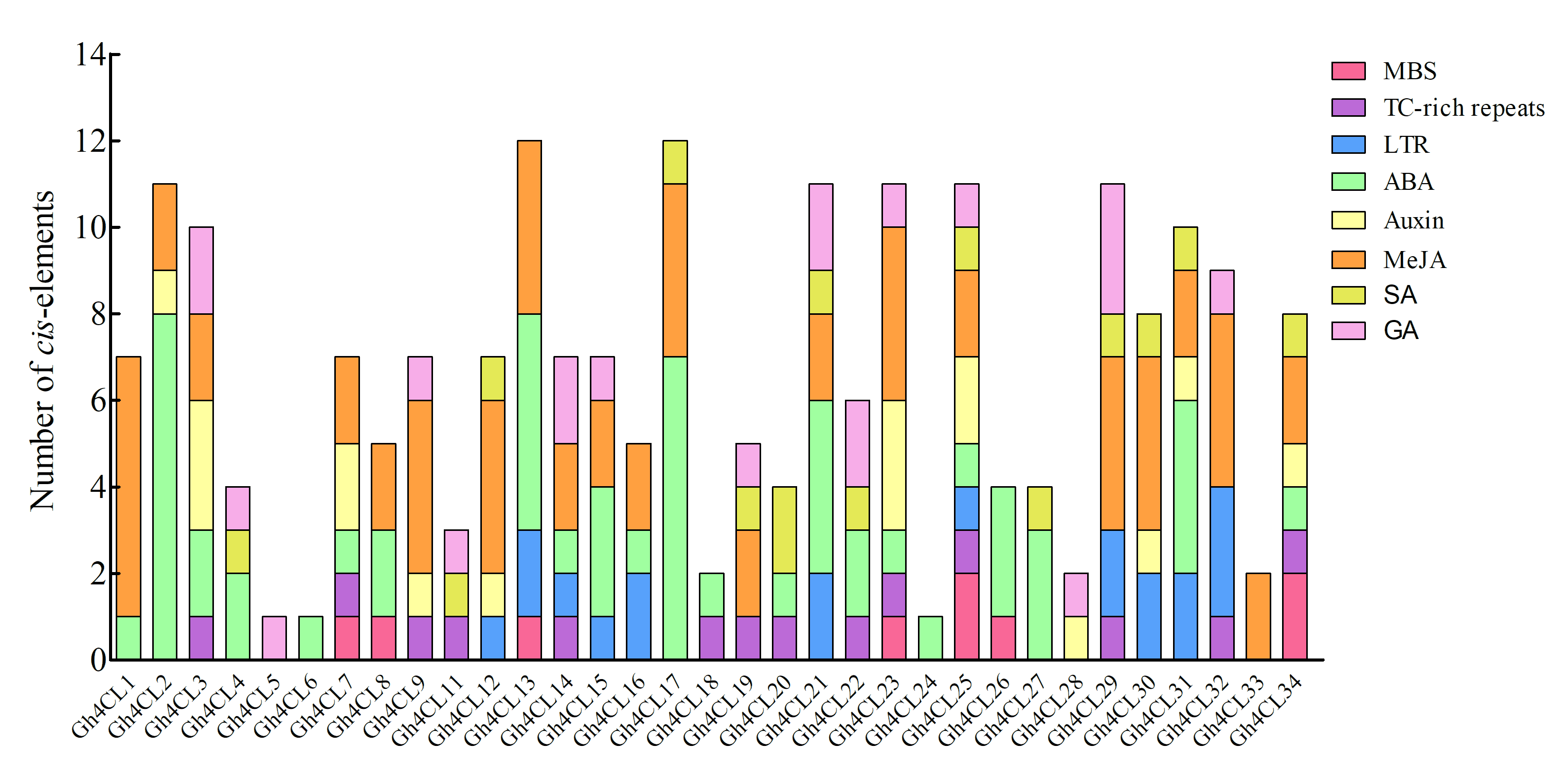


Fig. S3


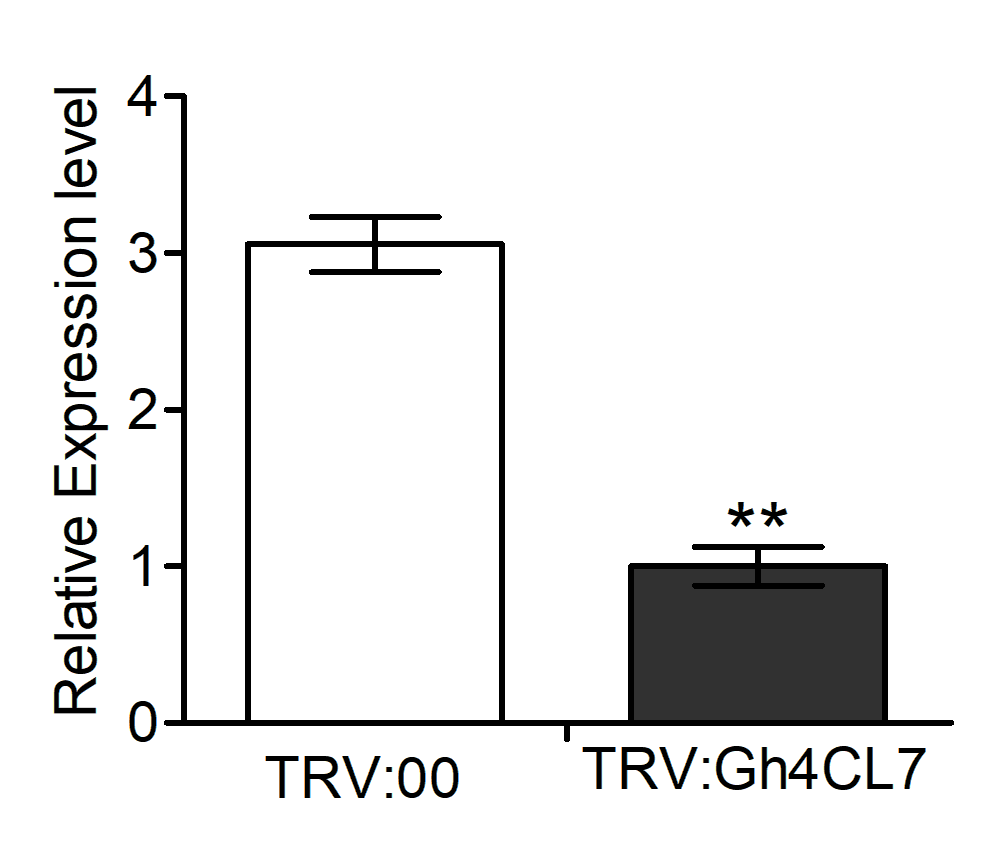


Fig. S4


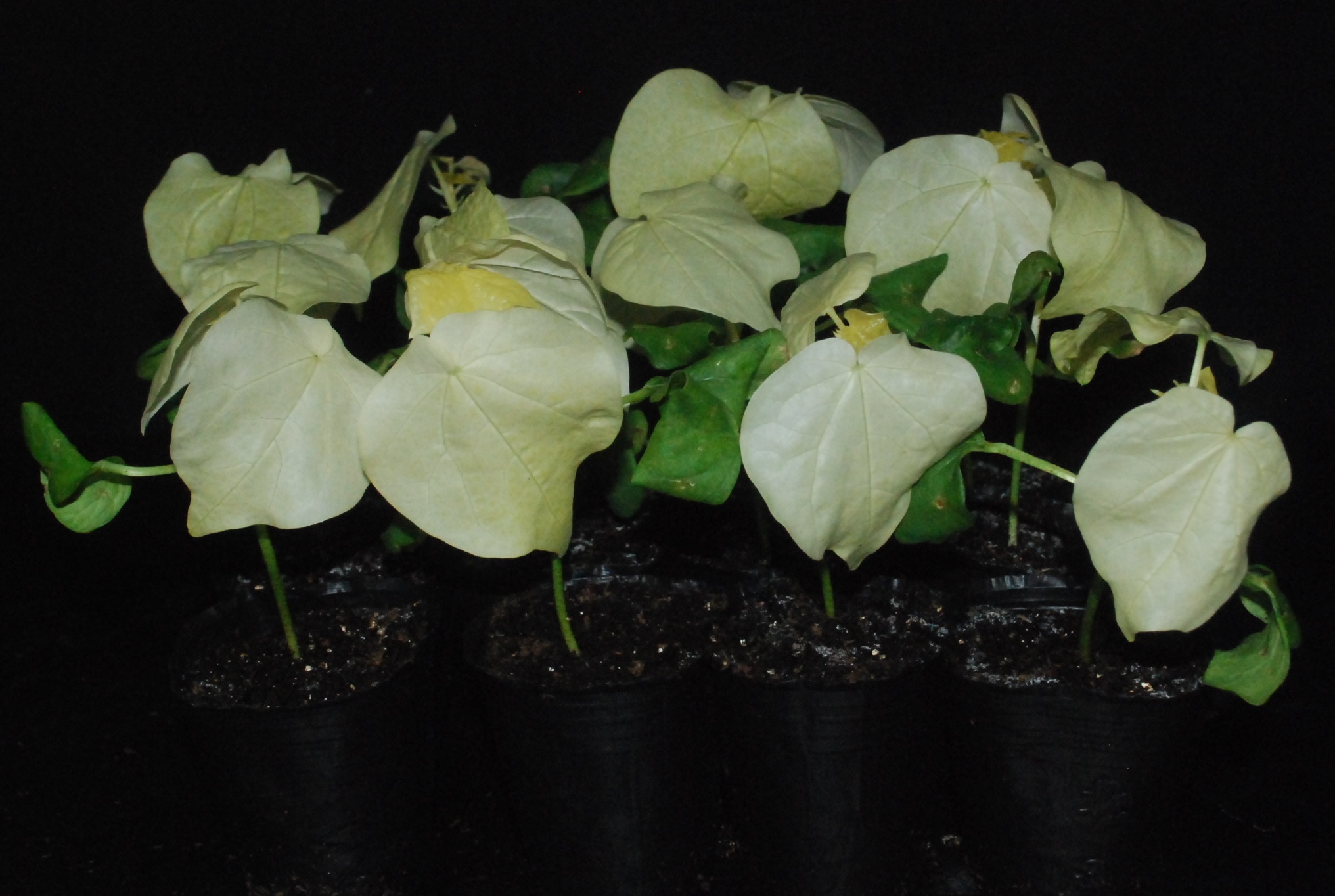


Fig. S5


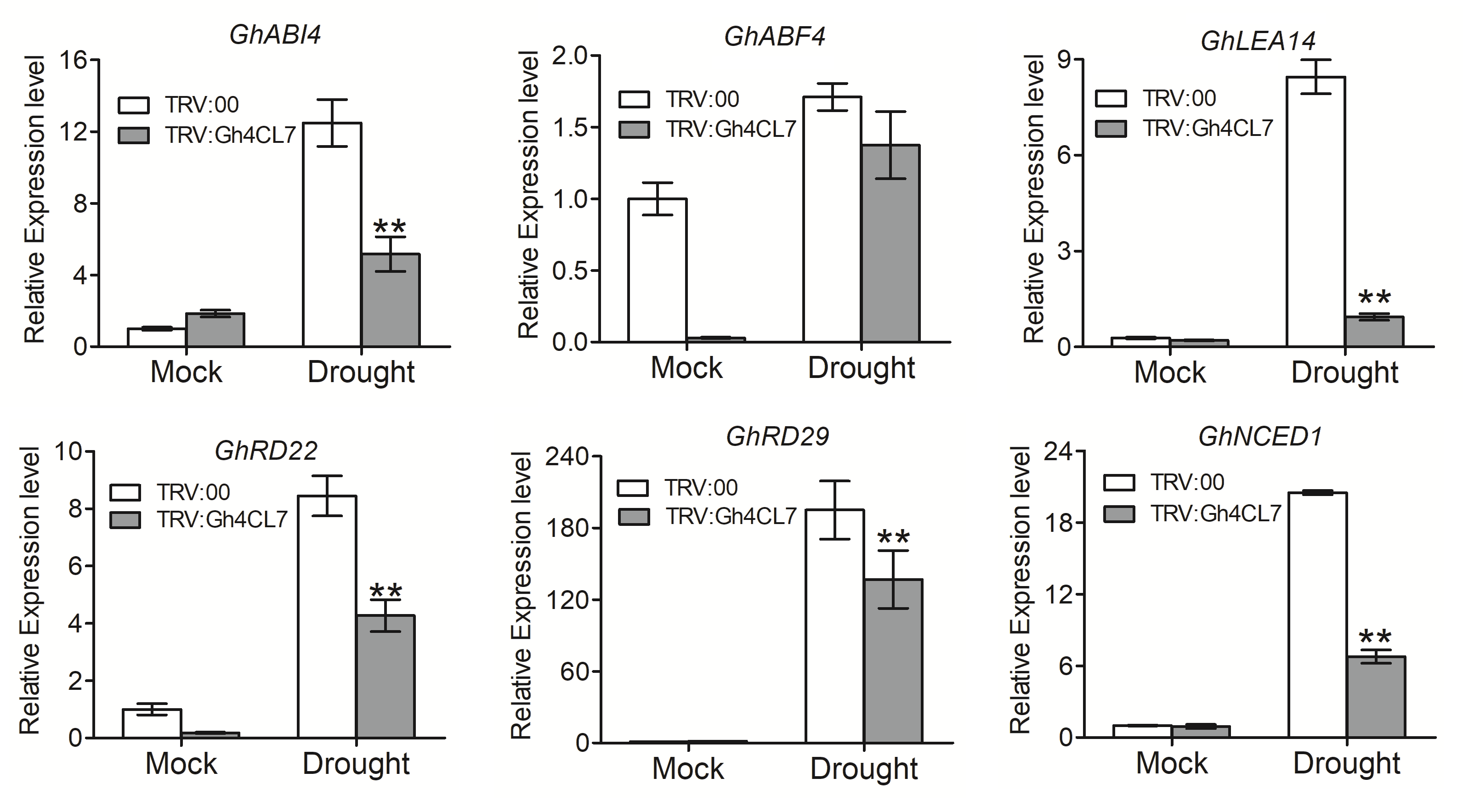


Fig. S6


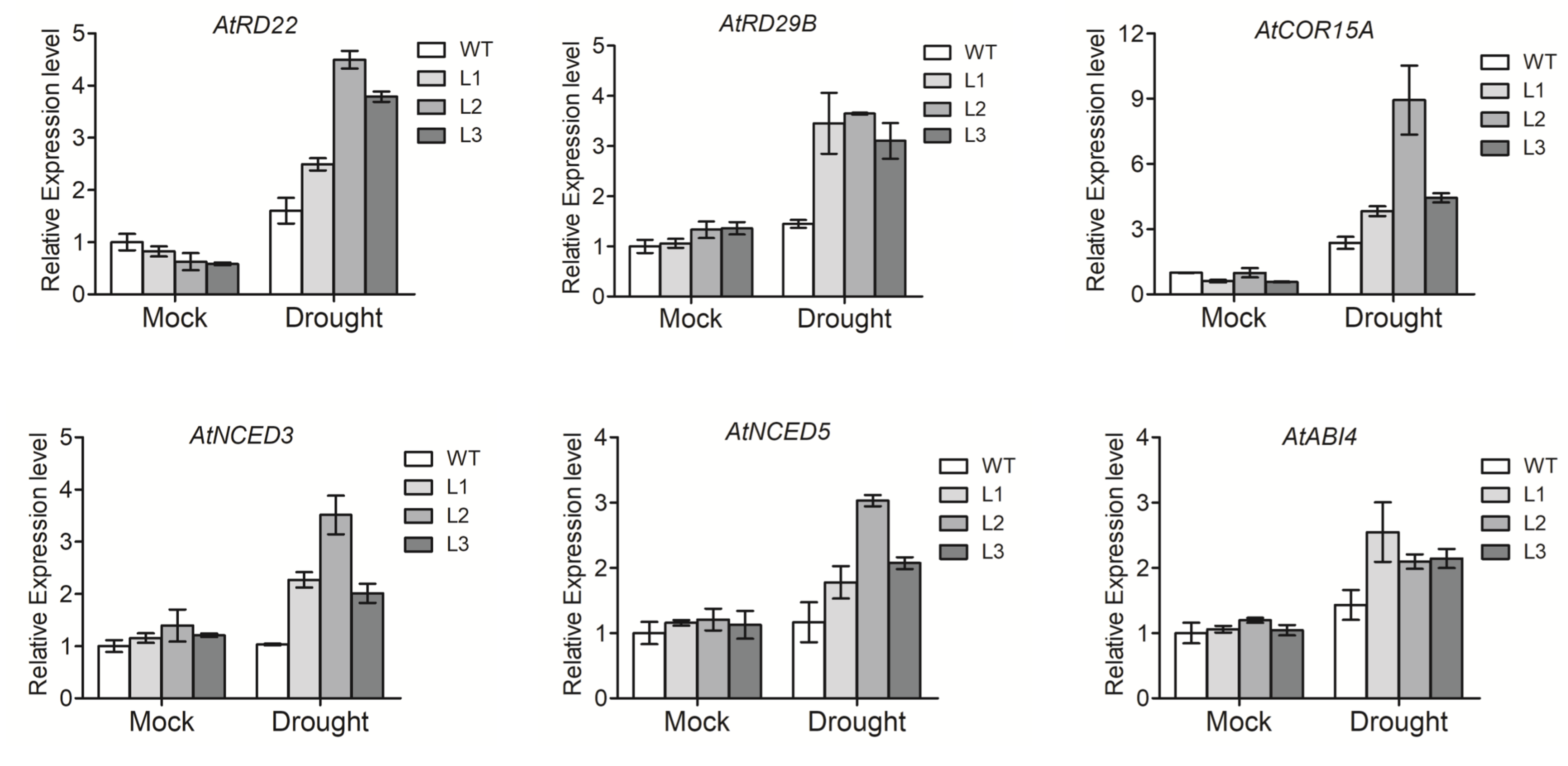
 Fig. S7

Supplement: Supplementary file 1 — Additional file 1: Figure S1. Gene structure and motif pattern of the Gh4CL genes. a Structural analysis. The left panel shows the neighbor-joining phylogenetic tree based on the amino acid sequences of the Gh4CLs. The classes I, II and III were marked correspondingly. The right panel shows the exon-intron structure of each Gh4CL gene with exons showing in orange boxes, introns in black lines between exons, and upstream/downstream UTRs in blue boxes. The number indicates the phase of the corresponding introns. The length of the Gh4CL genes is indicted by the scale line at the bottom. b Motif analysis. The motif analysis was performed by the MEME suite. Twenty motifs were detected and are displayed in color coded boxes. The length of proteins is indicted by the scale line at the bottom. Figure S2. Alignment of multiple Gh4CL and selected At4CL domain amino acid sequences. Multiple sequence alignment was performed using Clustal X. Box I and Box II represent the two conserved domains of the Gh4CL proteins. Figure S3. Analysis of cis-elements in the promoter of the Gh4CL genes. The numbers of different cis-elements are presented in the form of bar graphs. Figure S4. Relative expression levels of Gh4CL7 in plants infiltrated with TRV:00 and TRV:Gh4CL7 (n = 5). Total RNA was extracted from leaves at 2 weeks post-infiltration. Transcript levels were determined by qRT-PCR using GhUBQ7 as control. Figure S5. Silencing of the endogenous magnesium chelatase subunit I gene (GhCHLI) in cotton through VIGS. The leaf bleaching phenotype was observed 2 weeks after infiltration in TRV: GhCHLI plants. Figure S6. Transcription levels of stress responsive genes in CK and Gh4CL7-siliencing cotton plants. The normal condition plants were used as controls. GhUBQ7 gene was used as an internal control. All the gene expressions were normalized to the corresponding transcript levels in CK plants at normal condition. All the data represent mean ± SE for three biological replications. Figure S7 [file 12870_2020_2329_MOESM1_ESM.doc]
