# Supplementary material for: Characterization of the Gh4CL gene family reveals a role of Gh4CL7 in drought tolerance
Source: BMC Plant Biol. 2020 Mar 23;20:125. doi: 10.1186/s12870-020-2329-2 (PMC7092558; doi:10.1186/s12870-020-2329-2)
Supplement: Supplementary file 2 — Additional file 2: Table S1. Ka, Ks, Ka/Ks values for the Gh4CL paralogous gene pairs. Table S2. Information of the 20 motifs of Gh4CL proteins. Table S3. The primers used in qRT-PCR analysis. [file 12870_2020_2329_MOESM2_ESM.doc]

**Table S1**

| *Gh4CL* gene pairs | Ka | Ks | Ka/Ks |
| --- | --- | --- | --- |
| *Gh4CL2/Gh4CL17* | 0.00484 | 0.03555 | 0.1361 |
| *Gh4CL3/Gh4CL19* | 0.01742 | 0.06074 | 0.2868 |
| *Gh4CL5/Gh4CL18* | 0.00791 | 0.02054 | 0.3852 |
| *Gh4CL6/Gh4CL24* | 0.01141 | 0.04291 | 0.2659 |
| *Gh4CL7/Gh4CL25* | 0.00969 | 0.03205 | 0.3023 |
| *Gh4CL8/Gh4CL26* | 0.03276 | 0.06351 | 0.5158 |
| *Gh4CL9/Gh4CL29* | 0.0145 | 0.06246 | 0.2321 |
| *Gh4CL11/Gh4CL28* | 0.27929 | 0.40474 | 0.69 |
| *Gh4CL12/Gh4CL30* | 0.00727 | 0.03994 | 0.1821 |
| *Gh4CL13/Gh4CL31* | 0.01301 | 0.04016 | 0.3239 |
| *Gh4CL14/Gh4CL32* | 0.0137 | 0.02113 | 0.6482 |
| *Gh4CL15/Gh4CL33* | 0.00968 | 0.02627 | 0.3685 |
| *Gh4CL23/Gh4CL34* | 0.00664 | 0.05574 | 0.1192 |

**Table S2**

| Motif code | P-Value | sequence | characteristic domain |
| --- | --- | --- | --- |
| 1 | 3.60E-78 | EAKIVDPETGESLPPNQRGEJWLRGPTIMKGYLNBPEATARTJDKDGWLHTGDJGYFDEDGFLYIVDRJKELIKYKGYQ | AMP-binding enzyme |
| 2 | 4.90E-53 | APAELEALLLSHPEILDAAVIPYPDEEAGZVPVAYVVRSPGSSJTEEDIKQFIAKQV | AMP-binding enzyme |
| 3 | 1.00E-33 | RVKVNQSDTAAJLYSSGTTGLSKGVMLTHRNLIAS | AMP-binding enzyme |
| 4 | 1.00E-22 | QKFDFGEMLRAIEKYKVTHAPVVPPIV | AMP-binding enzyme |
| 5 | 4.30E-33 | GAIATTANPLSTPSEISKQVKDSKPKLAFTTSZLVDKVKSLKHPTI | NO |
| 6 | 1.90E-18 | FPNVKLGQGYGMTETGGVAAMEL | AMP-binding enzyme |
| 7 | 4.30E-19 | KYDLSSLRMIGSGGAPLGKEL | AMP-binding enzyme |
| 8 | 2.70E-21 | RVAFIDSIPKSPSGKILRKEL | AMP-binding enzyme |
| 9 | 1.60E-26 | LGJSKGDVVLLLLPNSIEFPJVFLAVMSJ | NO |
| 10 | 5.40E-20 | DVILCTLPMFHIYGLVLILLA | AMP-binding enzyme |
| 11 | 1.10E-28 | FLFQNISSYPSKPALIBATTGKTLTYSZLVSTVRSLASGLR | NO |
| 12 | 5.10E-14 | ETGIFRSKRPPIPLPPH | NO |
| 13 | 3.80E-11 | EEEAKYGSVGTLAPN | AMP-binding enzyme |
| 14 | 5.70E-23 | CIDSPPEGCLHFSELTZABEN | AMP-binding enzyme |
| 15 | 4.90E-33 | RNQQGFBVKNTPKILSFHDLLDIAGBVTE | NO |
| 16 | 3.20E-20 | VAQQVDGENPNLYFH | AMP-binding enzyme |
| 17 | 1.50E-08 | PYKRIR | AMP-binding enzyme |
| 18 | 2.50E-23 | LLDSPEFLSFLTZSNIDGDII | NO |
| 19 | 6.80E-19 | SLMVTKDQELAGDIH | AMP-binding enzyme |
| 20 | 1.3E-11 | QLRVGNAJVIM | AMP-binding enzyme |

**Table S3**

| Gene name | Genebank  number | Forward Primer 5’-3’ | Reverse Primer 5’-3’ |
| --- | --- | --- | --- |
| *AtRD22* | NM_122472 | GACTTTCGATTTTACCGACGAG | CGCTACCGGTTTTACCTTTATG |
| *AtRD29B* | NM_124609 | GAAACCAAAGATGAGTCGACAC | TTTTTCGTAAACCGGAGTCAAC |
| *AtABI4* | NM_129580 | GACTTCGTTTCATCATGAGGTG | AGTTCAAATCCTCCATCGAACT |
| *AtCOR15A* | NM_129815 | CATTAGCAGATGGTGAGAAAGC | TCTCAGCTTCTTTACCCAATGT |
| *AtNCED3* | NM_112304 | GATGAATTTGTTCCAGAGAGCG | AACACTAGGATCAGCCGTTTTA |
| *AtNCED5* | NM_102749 | CGACCGGTTATTAGCTATGTCT | TCTTCCGATGGTCTCTAAATCG |
| *AtEF_Lα* | NM_125432 | AACGGTGCCAGTGGGACG | CCTTGACAGCAACATTCTTGACAT |
| *GhRD22* | XM_016894800 | ACACACCGGACACAAGGGAAAG | AGCCACATTCGGATCGTCATGG |
| *GhRD29B* | XM_016869634 | AACTCCGGCAGAAACCATGGAC | TCTGCAATAACAGAGGTCGCCG |
| *GhABF4* | [XR_001686376](https://www.ncbi.nlm.nih.gov/nucleotide/XR_001686376.1?report=genbank&log$=nucltop&blast_rank=1&RID=ZPMK66RM014) | TTGAGTGGGGTTCCGCCATTTT | GTCTGGGGCAACTCTGTCATCC |
| *GhNCED5* | [XM_016832666](https://www.ncbi.nlm.nih.gov/nucleotide/XM_016832666.1?report=genbank&log$=nucltop&blast_rank=1&RID=ZPMR125Z016) | TATGGAACGCTTGGGAAGAGCC | CCGAGATTATTGGGCGACGTGT |
| *GhUBQ7* | DQ116441 | GAAGGCATTCCACCTGACCAAC | CTTGACCTTCTTCTTCTTGTGCTTG |
| *Gh4CL7* | MN897792 | TGCTGCTGTGGTCGGAATGAAA | AGAATTTTTCCCGACGGTGCCT |
| *Gh4CL8* | MN897793 | TCTCTCCCTTTTCCAGCGGACT | TTCCCAAAGACAGCAAGGCGAA |
| *Gh4CL11* | MN897796 | ATAAATCCAGGCAACCCAACAGG | TGGAACATTGAACTGGCTAACTT |
| *Gh4CL12* | MN897797 | CGACATCGGCCTACTGTTGGAG | GCACCGGACTTCAACATCCTCA |
| *Gh4CL13* | MN897798 | TCCAACTCGGAAATCGCTCACC | AAAATGGCTGCCGAATCGGACT |
| *Gh4CL17* | MN897802 | CCCAGTTGCATACGTTGTTCGC | GTCGACGTCGTTGCTTCTGGAT |
| *Gh4CL22* | MN897807 | GCTTGTTGTCACCGTCCCTGAA | ACCGACTTTGGGACACTGCTTA |
| *Gh4CL24* | MN897809 | GTAACCATTGCCCCATTCGTGC | CACCGGACATCACCATCCGAA |
| *GhPAL* | NC_030092 | CCTGGGTCAATCTTTGCTTC | AGGTCTCACCACCGAGTTTC |
| *GhCOMT1* | NC_030076 | CTTCCTGATTACCCCGACC | TAATTCCAGAAAATCCACCTTT |
| *GhCOMT2* | NC_030076 | CTCCCAGATTACCCAGATCC | ACCTTTGAGACAATCGCCC |
| *GhCOMT3* | NM_001326760 | GGCGAGATATGTTTGAAAGTGT | TGTGATGCTTTCTCAAATAGGC |
| *GhCCoAOMT* | FJ848871 | AAGAAGGGCCTGCAATGCCAGTT | GGTAACGGTGGTTCATTTGAGGCGA |
| *GhCCR1* | XM_017767698 | AGGATTGTTGATGACGCCTGAC | GTAGATTCTCGCCTTCTCCCAAC |
| *GhCCR2* | XM_016889406 | ATTGGCATCCATTTGGAGGAATG | ATGAGGAGACCAGGGCAGACAG |
| *GhCAD* | XM_016893894 | CCGTCATCAGCACTTCTCCGTC | CCCACAACTACCAAAGTCCCATT |
